# CA 15.3-driven F-18-FDG PET-CT for early detection of breast cancer recurrence: Diagnostic accuracy and clinical impact

**DOI:** 10.6026/973206300220107

**Published:** 2026-01-31

**Authors:** Sharma Neeraj, Singh Pritam, Banipal Raja, Brar Ripudaman

**Affiliations:** 1 Department of Nuclear Medicine, Guru Gobind Singh Medical College & Hospital, Faridkot, Punjab, India; 2 Department of Radiation Oncology, Government Medical College, Patiala, Punjab, India

**Keywords:** Breast cancer, CA 15.3, PET-CT, recurrence, negative conventional imaging, clinical management

## Abstract

An isolated rise in serum tumor marker CA 15.3 during post-treatment surveillance of breast cancer patients represents a notable diagnostic challenge. In many such cases, conventional imaging modalities fail to identify occult sites of early disease recurrence. Consequently, this diagnostic limitation may result in a delay in timely and appropriate clinical management. This study aimed to evaluate the diagnostic accuracy of whole-body F-18-FDG PET-CT imaging in early localization of breast cancer recurrence in patients with elevated CA 15.3 levels. Hence, the study included 50 routine breast cancer patients at Guru Gobind Singh Medical College & Hospital, Faridkot, who showed signs of disease relapse and were monitored for signs of recurrence, based on rising tumor markers. This study found a significant correlation between elevated CA 15.3 levels and PET-CT-detected recurrences in patients. Bone recurrences had the highest combined CA 15.3 levels. PET-CT findings altered clinical management in 80% of cases, with 28/40 receiving targeted radiotherapy, 9/50 initiating new systemic therapy and 3/40 starting with endocrine therapy. PET-CT is essential for the early detection of occult recurrences in breast cancer patients with elevated CA 15.3 levels.

## Background:

Breast cancer continues to be the most prevalent disease diagnosed in women worldwide and is a major contributor to cancer-related death [1]. Remarkably, the death rate has declined over the past few decades despite an increase in the tumor's occurrence. This is ascribed to advancements in increased systemic and local therapy as well as early tumor diagnosis through screening techniques [2, 3]. Up to 30% of patients experience recurrence, therefore continues a severe therapeutic problem despite significant improvements in early identification and treatment. Recurrence identification must be done promptly and accurately since it has a direct impact on long-term survival and treatment planning [4]. Conventional imaging modalities are essential for routine surveillance. Still, they are not always effective in locating the disease, particularly in cases of relapse in previously treated patients, whose distinction between post-surgical changes and recurrence may be exceedingly challenging [5]. Nevertheless, their capacity to identify early or small-volume metastatic disease may be restricted. In this context, serum tumor markers, particularly Cancer Antigen 15.3 (CA 15.3), have drawn attention as a post-treatment surveillance tool. In patients with metastatic or recurrent breast cancer, CA 15.3, a high-molecular-weight mucin-like glycoprotein that is associated with the MUC1 gene, is frequently elevated. A potential early warning biomarker, a rising CA 15.3 level can precede radiological evidence of recurrence by several months, as reported by numerous studies [6]. However, CA 15.3 is incapable of detecting anatomical localization independently, and its specificity may be influenced by non-malignant conditions or other tumor types. As a result, the effectiveness of this serum tumor marker is substantially enhanced when it is employed in conjunction with a sensitive imaging modality. F-18-fluorodeoxyglucose positron emission tomography fused with computed tomography (FDG PET-CT) has evolved into a crucial whole-body imaging modality for identifying metabolically active tumors. Research indicates that FDG PET-CT exhibits superior sensitivity and specificity compared to conventional imaging in detecting recurrent breast cancer, especially in instances with ambiguous radiologic findings [7, 8]. Whole body PET-CT has proven effective in patients who have elevated tumor markers but without radiology signs of disease, facilitating early detection of recurrence and impacting clinical therapy decisions. The role of PET-CT in monitoring asymptomatic patients with isolated CA 15.3 elevation is still debated, despite growing evidence and lacks universally accepted guidelines. Clinicians often face uncertainty regarding the appropriate timing for initiating PET-CT during the follow-up period, particularly in cases where patients' exhibit elevated CA 15.3 levels alongside negative conventional imaging results [9]. Therefore, it is of interest to report the role of F-18-FDG-PET-CT in assessing patients with treated breast cancer who exhibit rising tumor markers and negative conventional imaging, focusing on the early localization of any recurrence and its impact on clinical management decisions.

## Materials and Methods:

A total of 50 routine patients of Guru Gobind Singh Medical College & Hospital, Faridkot, who were previously diagnosed and treated for breast cancer and were on follow-up, and who showed any clinical or biochemical suspicion of disease relapse, were included in the study to facilitate the early localization of recurrence. The mean ± SD of the patients was 49.9 ± 10.2 years, with a range of 30-65 years. The study was carried out from November 2015 to November 2017. The histopathology distribution of each patient was recorded, with IDC 46/50 (92%) and ILC 4/50 (8%). The primary tumor location for all patients was determined to be UOQ-18/50 (36%), UIQ-12/50 (24%), LOQ-11/50 (22%), and LIQ-9/50 (18%). The tumor size at initial staging was recorded as (mean ± SD: 3.5 ± 2.3 cm, range: 1.1-10.2 cm). Additionally, the serum tumor marker CA 15.3 and baseline PET-CT of all 50 patients were recorded. All patients received primary treatment following diagnosis, which included surgery, chemotherapy, and radiotherapy based on the clinical scenario. 28/50 (56%) of participants, received hormonal therapy following primary treatment. After treatment, patients went through routine follow-up with serial measurements of the tumor marker CA 15.3 at intervals of 3 to 6 months over a period of 1 to 2 years. In the follow-up of breast cancer cases, patients with diffuse body pains, fever, or shivers, as well as negative or inconclusive radiological imaging and/or rising tumor marker levels (>15%) were considered for whole body PET CT imaging to detect any recurrence. The CA 15.3 tumor marker blood test was carried out in the Department of Biochemistry using the chemiluminescence method on a fully automated sample analyzer and whole body PET CT imaging was performed on a dedicated PET-CT unit, which comprises a 16-slice CT, in the Department of Nuclear Medicine at Guru Gobind Singh Medical College and Hospital, respectively. Standard protocol of whole body F-18-FDG PET-CT imaging was followed. 10mCi dose of F18-FDG was injected I/v, after overnight fasting and scan was taken 60min post injection. Oral and I/v contrast agents were only administered in patients who presented clinical symptoms regarding the same to ensure diagnostic CT data. The images were visually evaluated by two experienced nuclear medicine physicians and in case of need; a viewpoint of radiologist was also taken at times to reach a final diagnosis. The majority of malignant cancer phenotypes demonstrate elevated glucose metabolism. Glucose is taken up by tumor cells by facilitated transport and then undergoes glycolysis. F-18-fluoro-2-deoxyglucose (F-18-FDG) being a glucose analog is readily taken up by the malignant cells using facilitated transport similar to that used by glucose, thus gets incorporated inside the cells for the normal glucose metabolism and CO2 and H2O diffuse out from the cell whereas F-18 gets trapped in the cells [10]. The radioactive glucose localizes in malignant cells six times more than the normal cells and facilitates to diagnose malignancy or metastasis by PET-CT. This unique property of radiopharmaceutical F-18-FDG enables to detect cancer reoccurrence with the help of whole body PET-CT imaging. Recurrence in PET-CT was assessed by a focally increased glucose uptake using the maximum 'standard uptake value' (SUVmax) as a reference [11]. Any FDG uptake deviating from physiological distribution was regarded as an abnormal finding. The ethical clearance has been taken from the institutional ethical committee vide reference no BFUHS/Ex/PHD/E15/719

## Statistical analysis:

Correlations between CA 15.3 and SUVmax were assessed using Spearman's rank correlation (ρ). Group comparisons (e.g., ER+ vs. ER-tumors) employed Mann-Whitney U tests. Multivariable linear regression analyzed SUVmax predictors (CA 15.3, tumor subtype, recurrence site). Analyses used [SPSS software] with *p* < 0.05 considered significant.

## Results:

The study included 50 breast cancer patients (mean age: 50.02 ± 10.1 years) with suspected recurrence based on rising CA 15.3 (>30 U/mL) and negative or inconclusive conventional imaging. The clinical characteristics of all selected patients are shown in Table 1. Histopathological analysis revealed invasive ductal carcinoma (IDC) in 92% (46/50) and invasive lobular carcinoma (ILC) in 8% (4/50). Primary tumors were predominantly located in the upper outer quadrant 18/50 (UOQ; 36%), followed by upper inner 13/50 (UIQ; 26%), lower outer 10/50 (LOQ; 20%), and lower inner quadrant 9/50 (LIQ; 18%). Mean tumor size at diagnosis was 3.3 ± 2.2 cm (range: 1.1-10.2 cm), with 62% (31/50) being ER/PR-positive and 30% (15/50) HER2-positive. 9/50 (18%) were triple negative.

The distribution of recurrence localizations in PET-CT findings are shown in Table 2. PET-CT detected recurrence in bone = 12, chest wall = 10, extra axillary lymph node area = 9, liver = 5, lung-pleura = 4, pelvis-peritoneum = 5, regional-local = 5, with a mean SUVmax of 7.4 ± 2.0 (range: 4.2-13.5).

**Figure 1:**
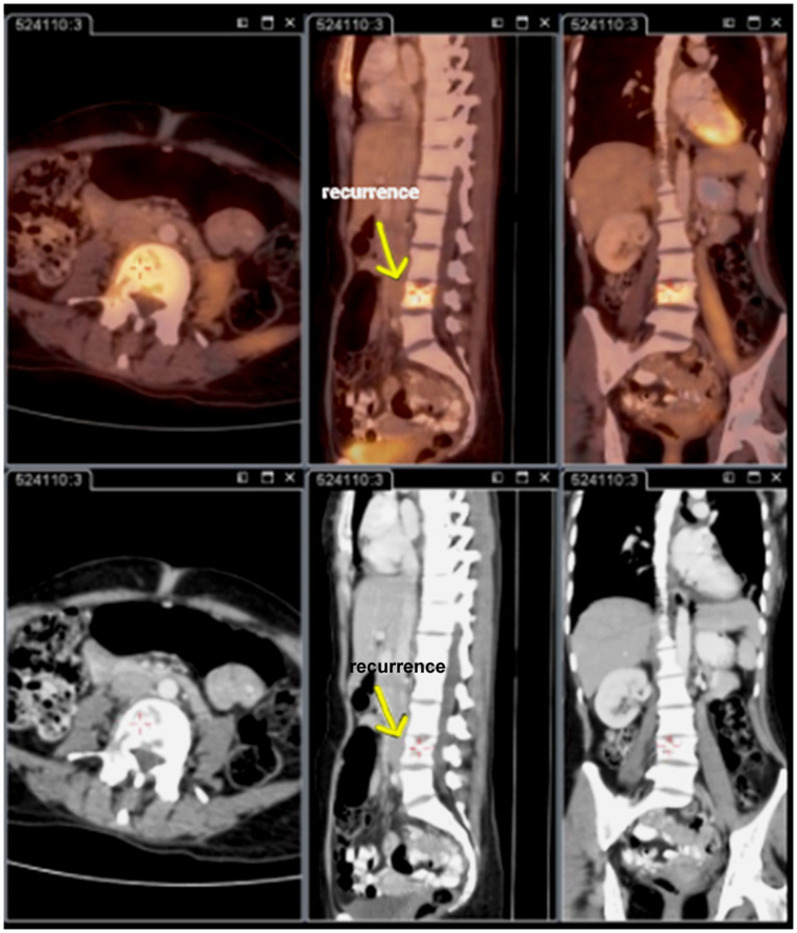
Whole body PET-CT performed for isolated CA 15-3 elevation (45-year-old female, 2-years post treatment) revealed an FDG avid lumbar spine lesion (arrow), confirming recurrence in bone and leading to a change in treatment strategy.

Bone was the most prevalent site of recurrence, 24% of the cohort and a mean SUVmax of 9.4 ± 1.9 (range: 6.9 -12.8) followed by chest wall, 20% exhibiting a mean SUVmax of 5.8 ± 0.5 (range: 5.1-6.7). This site exhibited reduced metabolic activity. Recurrence was also observed with intermediate metabolic activity in extra-axillary lymph node area (18%), regional/local (10%) and lung/pleural location 4/10 (8%) with mean SUVmax: 6.5 ± 1.1; range: 4.2-7.8, SUVmax 6.4 ± 0.7; range: 5.8-7.3and SUVmax 6.7 ± 0.8; range: 6.1-7.9 respectively. In 5/50 (10%), liver recurrence was observed, and the metabolic activity range was the widest (mean SUVmax: 9.2 ± 2.8; range: 6.3-13.5). In 5/50 (10%), pelvic/peritoneal involvement was observed, with relatively consistent SUVmax values (6.3 ± 0.5; range: 5.5-6.8). This distribution indicates bone as both the most frequent and metabolically active site of recurrence, as shown in Figure 1 in which a 45-year-old patient, after primary treatment was on follow up for 2 years, considered for whole body PET-CT assessment after elevated CA 15-3 serum levels and a focal lesion was seen in lumbar spine demarks clear case of recurrence, followed by distinct patterns of metabolic activity in other anatomical locations.

Patients with ER/PR-positive tumors demonstrated significantly elevated SUVmax values (8.2 ± 2.3 compared to 6.5 ± 1.8 in ER/PR-negative tumors; p=0.02), indicating increased metabolic activity in hormone receptor-positive illness. Elevated CA 15.3 levels showed a significant correlation with PET-CT-detected recurrences in this study. Among this study patients with PET-CT-positive findings (n=50), the mean CA 15.3 level was 66.5 ± 23.1 U/mL Site-specific analysis revealed that bone recurrences demonstrated the highest combined CA 15.3 levels (94.7 ± 18.5 U/mL) followed by chest wall (62.7 ± 23.1), extra-axillary lymph node area (57.4 ± 17.2), regional/local (48.1 ± 7.5), liver (52.5 ± 11.9), pelvis/peritoneum (64.7 ± 7.7), and lung/ pleura region (54.3 ± 12.7). PET-CT findings directly altered clinical management in 80% of cases (40/50): (28/40) received targeted radiotherapy along with systemic therapy for isolated recurrence sites, (9/50) initiated new systemic therapy and (3/40) started with only endocrine therapy.

## Statistical findings:

Spearman's correlation revealed significant associations between CA 15.3 levels and metabolic activity across recurrence sites, with strongest correlations observed in bone metastases (ρ = 0.72, *p* < 0.001) and liver lesions (ρ = 0.65, *p* = 0.002). ER/PR-positive tumors demonstrated markedly higher SUVmax compared to ER/PR-negative tumors (8.2 ± 2.3 vs. 6.5 ± 1.8; *p* = 0.02).

## Discussion:

Recurrent breast cancer can pose considerable diagnostic and therapeutic challenges for the oncologic team. The period of 1 to 3 years is very crucial for CA breast patients post treatment completion. Early localization and accurate restaging of recurrent breast cancer is important to the selection of the most appropriate therapeutic strategy, mainly by identifying patients with limited disease who could benefit from curative treatment. We examined a cohort of patients who were on routine follow up post treatment of breast cancer. This study demonstrates the critical role of whole body PET-CT in detecting occult breast cancer recurrence in patients with rising CA 15.3 levels and negative conventional imaging. Our findings highlight bone as the most frequent (24%) and metabolically active (mean SUVmax 9.4 ± 1.9) site of recurrence, followed by the chest wall (20%) and extra-axillary lymph nodes (18%). The association between elevated CA 15.3 levels and bone recurrence is well-documented, with studies reporting specificity >75% for CA 15.3 >80 U/mL in bone-dominant disease [12]. Our findings further validate this correlation, as bone metastases in our cohort exhibited the highest mean CA 15.3 levels (94.7 ± 18.5 U/mL). CA 15.3 serves as a surrogate marker for early localization of recurrence, particularly when levels rise >25% from baseline as observed by Nicolini *et al.* [13]. This aligns with our observation that whole body PET-CT-findings detected lesions correlated with the rising serum tumor marker CA 15.3, emphasizing the biomarker's role in early detection.

The positive correlation between CA 15-3 and SUVmax (r = 0.72, p < 0.001) underscores the potential utility of serial CA 15-3 monitoring in identifying recurrence before it becomes clinically overt. These findings are consistent with previous studies, such as those by Mwania *et al.* [14]. Consistent with prior evidence; whole body PET-CT outperforms conventional imaging in the early detection and localization of recurrences, particularly in bone and visceral body [15, 16]. Our findings reinforce its pivotal role in evaluating rising CA 15.3, where it identified recurrence in 100% of cases with negative conventional imaging. The higher SUVmax in ER/PR-positive tumors (8.2 ± 2.3 vs. 6.5 ± 1.8; p=0.02) suggests that hormone receptor-positive recurrences may exhibit greater glycolytic activity, possibly reflecting aggressive biological behavior in advanced stages [17]. The liver, despite fewer cases (10%), showed the widest SUVmax range (6.3-13.5), underscoring the need for vigilant surveillance in high-risk patients with rising CA 15.3.HER2-positive recurrences in our cohort demonstrated intermediate metabolic activity, consistent with reports of variable FDG uptake in this subtype [18] underscoring the need for receptor- specific interpretation of PET/CT findings. The clinical impact of our PET-CT findings (altering management in 80% of cases) supports Ulaner *et al.* [19] conclusion that quantitative PET parameters provide critical prognostic information in recurrent breast cancer. Additionally, our results showing a critical clinical impact 24/40 received targeted radiotherapy along with systemic therapy for isolated recurrence sites, also aligns with Filippi *et al.* [20] who demonstrated PET-CT's pivotal role in guiding therapy for tumor marker-positive recurrence, with radiotherapy/surgical options pursued in 26% of cases despite negative conventional imaging. However, the lower SUVmax in chest wall recurrences (5.8 ± 0.5) highlights the importance of combining metabolic and anatomic imaging to avoid false negatives. Moreover, the National Comprehensive Cancer Network (NCCN) [21] now includes PET-CT as standard for evaluating rising CA 15-3, particularly given its ability to identify isolated recurrences amenable to targeted therapy - a pattern observed in 70% of our radiotherapy-eligible cases.

## Limitations:

Our study is limited by its single-center design and small sample size, particularly for HER2-positive and ILC subtypes. Larger cohorts are needed to substantiate CA 15.3 thresholds for site-specific recurrence forecasting.

## Conclusion:

PET-CT plays a vital role in the early detection of occult recurrences in breast cancer patients exhibiting elevated CA 15.3 levels and inconclusive conventional imaging. The findings provide substantial evidence for the integration of PET-CT as a standard component of follow-up care in high-risk patients. Monitoring CA 15.3 levels assists clinicians in determining which patients require PET-CT scans, thereby facilitating improved treatment decisions, particularly for those who may benefit from targeted radiation or modifications to their medication regimen.

## Figures and Tables

**Table 1:** Clinical characteristic of breast cancer patients (n-50) with suspected recurrence

**Characteristics**	**n (%)**	**Mean (range)**
Age at diagnosis	50	50.02 (30 - 65)
**Histology**		
Invasive Ductal Carcinoma (IDC)	46 (92%)	
Invasive Lobular Carcinoma (ILC)	4 (8%)	
**Primary tumor location**		
UOQ	18/50 (36%),	
UIQ	12/50 (24%),	
LOQ	11/50 (22%),	
LIQ	9/50 (18%).	
**Hormonal Status**		
ER/PR-positive	31/50 (62%)	
ER/PR-negative	19/50 (38%)	
HER2-positive	15/50 (30%)	
Triple negative	9/50 (18%)	
**pTNM staging**		
pT1N0M0	8 (16%)	
pT2N0M0	14 (28%)	
pT2N1M0	20 (40%)	
pT3N1M0	6 (12%)	
pT3N2M0	2 (4%)	
Value of CA 15.3 at the		66.5 (36.0 - 118.9)

**Table 2:** Distribution of recurrence localizations in PET-CT findings

**Recurrence localization**	**Number of patients (%)**
Bone	12 (24%)
Chest wall	10 (20%)
Extra-axillary lymph node area	9 (18%)
Liver	5 (10%)
Lung & pleura	4 (8%)
Pelvis & peritoneum	5 (10%)
Regional & local	5 (10%)
